# LBS user location privacy protection scheme based on trajectory similarity

**DOI:** 10.1038/s41598-022-18268-8

**Published:** 2022-08-17

**Authors:** Kun Qian, Xiaohui Li

**Affiliations:** School of Electronics and Information Engineering, Liaoning University of Technology, Jinzhou, 121001 China

**Keywords:** Engineering, Mathematics and computing

## Abstract

During the data set input or output, or the data set itself adds noise to enable data distortion to effectively reduce the risk of user privacy leakage. However, in the conventional method, the added noise may cause data distortion, thereby appealed against it. However, the amount of noise is too small and cannot meet the effect of privacy protection. Therefore, we propose a LBS user location privacy protection scheme based on trajectory similarity (DPTS). With double privacy protection without reducing the efficiency of algorithms, it does not cause data distortion to provide more reliable privacy protection. The main contributions of this article include: (1) In the process of collecting and publishing the location data, introduce into the privacy protection method, (2) The differential privacy algorithm based on the trajectory prefix tree is superimposed on the basis of the false position replacement algorithm based on the trajectory similarity, (3) Propose LBS-based Difference Privacy Protection Algorithm. In the algorithm, We reach the purpose of protecting user personal privacy by replace the original trajectory into a fake track trace that is the lowest degree of similarity in the interval. Then establish a prefix tree and add noise to the positional frequency. It is in order to further protect the sensitive location information, double protection in the trajectory data set, and the degree of privacy protection is improved. Simulation experiment results show that the proposed algorithm is effective. The algorithm can suppress the distortion rate of data while improving the amount of noise, and in improving the algorithm operation efficiency, it reduces the risk of leakage of sensitive position information.

## Introduction

In order to adapt to the intelligence of personal consumer demand, mobile application software has become more popular in recent years, and most mobile phone software and navigation systems use location services^1,2^. Location services have penetrated into the square of people's lives. Location service has penetrated into all aspects of people's lives. In terms of traffic travel, such as Auto Navi Map, Baidu Map. In terms of living, such as Meituan-Dianping Takeout, ELEME Takeout, Trip.com Group, etc. Users can search the nearby location information by these LBS applications to select the service data they need. The popularity of location service applications makes people's daily life more convenient and fast, but the location information is very likely to reveal a wide variety of personal privacy or sensitive information, the attacker may further Analysis of users' home address, work units, hobbies and daily action routes, etc. Then use this information to recommend the advertisements they are interested in, even fraud or track them. Therefore, protecting the privacy of users has become a popular research direction.

In recent years, many research results have been made in the direction of location privacy protection, most of which use encryption technology, multi-party safety calculations, federal learning, differential privacy and other methods^3–7^. Although the effect of privacy protection has reached a certain extent, there are still many vulnerabilities and imperfections. For example, the literature^8^ proposes a position K-Anonymity model, a K-Anonymity algorithm, generating (k − 1) an anonymous user to override the original track and location data, and protect the original location of the query user; In the literature^9^, users use their original location to store buffers and randomly generate K − 1 fake positions within the defined range. When the user issues a service request to the LBS, the system sends the buffer including the original position and the K position thereof to the LBS server to achieve K-Anonymity user trajectory.

However, because the fake position is randomly generated, there may be many restrictions or obvious omissions in the fake position, so that the attacker can see it at a glance that it is forged. Literature^10^ has improved this point. The sample space of each original sample is specified in this literature, and the grid is used to divide it, and then the query probability is calculated according to the query history of each grid space. Combined with the query probability elects a hierarchical (k − 1) pseudo position with the historical query probability similarity of the original location of the user. This effectively avoids the attack of the attacker's obvious query probability between the original position and the fake position, and it is a fake.

But there are still problems such as location semantic differences. Literature^11^ in response to this issue, first uses the “geographical irresponsible” framework to build a expected distance. Construct the semantic location information by defining privacy quality functions and requirements functions to determine the sensitivity of different position points. Finally, the Laplace noise is added according to the sensitivity of the position point to different types of regional regional fine particle size, which systematically solves the contradiction between positioning privacy protection, service availability and time over time.

However, due to a single use of K-anonymous or false positions, sometimes it is not possible to protect the user's personal information and sensitive location information, so many trajectory privacy protection models still have a lot of defects and attacks. It is still impossible to solve the problem that when the trajectory information is involved in the starting location and the termination location, the attacker is recognized due to the obvious changes in the trajectory information. With the gradual maturity of differential privacy technology, many researchers have been selected to solve privacy leaks. Although it can effectively ignore the background knowledge of the attacker, it will cause data distortion due to excessive adding noise, and too little can not meet the effect of privacy protection. So still need researchers to continue to explore better improvements.

Literature^12^ using digital signature technology to analyze the solution based on pseudonym technology and encryption algorithm. Overcoming privacy leaks and DOS attacks by using signed authentication and promises to promise. Use signature authentication to reduce the effects of DOS and Sybil attacks. This can improve users' privacy and location-based IoT services security. In addition, a faster 5G solution is proposed, which can quickly spread data to fast-moving vehicles. This scheme can effectively solve the problem caused by attacks in any IoT environment.

Literature^13^ based on the evaluation of various online scenarios, a lightweight trust model was proposed. When meeting privacy and security requirements, a pseudo-name method is adopted to identify dishonest nodes in the MITM attack scene and revoke its credentials. This model adopts an encryption algorithm to more accurately identify whether the sender and the receiver of the information are invaders. The algorithm modules such as Authentication, Peer Alert Message, and Time Stamp Verification are further strengthened to strengthen privacy protection, thereby reducing the loss of information.

Literature^14^ presented an efficient pseudonym change strategy with multiple-mix zones scheme to provide trajectory privacy for road network. This scheme is composed of a computer platform with multiple mixed areas and a cheating detection mechanism. First of all, the PC has a multi-mixed area strategy, allowing vehicles to effectively change its pseudonym to realize the road network with trajectory dynamics. Secondly, in order to fight the cheating attack mechanism, a cheating detection mechanism was built. Allowing periodic verification pseudo-name changes to be successful, at the same time, malicious vehicles can also be detected. Through such a deception mechanism to protect vehicles from linked attacks, the protection of user trajectory privacy is achieved. This method can be widely used in road network user trajectory protection algorithms that require pseudonym, and can effectively solve the privacy vulnerabilities that vehicles change when they change pseudo-names in inappropriate occasions.

Because most of the existing privacy protection models only consider social location, they ignore the non-social location of vehicles during traffic signal lights and traffic jams. Literature^15^ proposed a new multi-mixed zone solving related privacy model for this issue. Considering the relationship between the parking location and the position of traffic lights and traffic jams, use non-related hybrid areas to replace the area where parking lots and traffic lights and traffic jam are located. This model can effectively solve the problem of privacy leakage of vehicle trajectory privacy caused by traffic lights or traffic jams caused by traffic lights or traffic jams.

The privacy protection of the trajectory information of the vehicle itself on the Internet of the Internet has matured. On this basis, this article proposes a dual privacy protection plan for user LBS based on trajectory similarity. The program combines a false location, frequent mode mining and differential privacy technology. The first is a trajectory similarity algorithm based on a false position. Replace the user's original location with the lowest possible position; Secondly use the location information tree data structure to make frequent mode excavation and effectively maintain the relationship between data items; Finally, use the Differential privacy to disturb the disturbance position frequency, improve data processing efficiency. Generalized sensitive location information, improve accuracy, reduce refusal rate.

## Relevant definitions

This section mainly explains the basic definitions and presentations related to the algorithm. Including trajectory similarity, TRIE tree, laplas' socking mechanism under differential privacy, etc. Table 1 lists some system parameters and meaning used in this article.

**Table 1:** System parameters and meaning.

Parameter	Meaning
t	time
(lati,lngi)	The latitude and latitude of the i query location
T	Trajectory
TU	Primitive trajectory collection
TC	Candidate trajectory collection
*ϕ*_*i*_	Candidate and primitive position relative to the movement of the movement trajectory of the initial time of *t*_*i*_ and *t*_0_
dis (TU,TC)	The Euclidean distance between the candidate and the original position
P_i_	Position access frequency
Fake (LID_i_)·*ω*	Fake(LID_i_)’s weights
σ^2^	Trajectory similarity
H(X)	Location information entropy
P(X)	Output probability function
Δ*f*	Global sensitivity of function *f*
${\text{Lap}}\left( {\frac{\Delta f}{\varepsilon }} \right)$	Laplace noise
*ε*	Privacy budget

### Trajectory

#### **Definition 1**

*(Track similarity*^16^*)* The trajectory similarity is a method of comparing the similarity of the two trajectories by the similarity of the position point change direction of the track similarity function metrics. It is basically defined as follows:

User's true motion trajectory is : Lu = {(x 0,y0,T0) , (x 1,y1,T1), … , (xn, yn, Tn)} ((xi, yi, Ti) indicates the geographic coordinates of the user at Ti time (xi, yi); lng and lat represent longitude and latitude, respectively; The sample time ti satisfies T0 < T1 < , …, < Tn)).

Suppose the direction of motion trajectory of the user at TI is changed to $\upphi {\text{i}}\sqrt {{\text{b}}^{2} - 4{\text{ac}}}$ relative to the direction of the initial T0. which is $\tan \phi i = ( {yi - y0} )/( {xi - x0} )$. That is $\upphi {\text{i}} = {\text{arc}}\tan ( {yi - y0})/( {xi - x0} )$. Then the user's original trajectory can be expressed as ${\text{Lu}} = \{ ( {{\text{x}}0,{\text{y}}0,{\text{t}}0} ),\left\{ {( {\Phi 1,{\text{t}}1} ),( {\Phi 2,{\text{t}}2} ), \ldots ,( {\Phi {\text{n}},{\text{tn}}} )} \right\}$. Similarly, the original trajectory corresponding to the original trajectory ${\text{Lc}} = \{ {( {x0,y0,t0} ),( {{\text{x}}^{c} 1,{\text{y}}^{c} 1,{\text{t}}1} ), \ldots ,( {{\text{ x}}^{c} n,{\text{y}}^{c} n,{\text{tn}}} )} \}$. Also equivalent to ${\text{Lc}} = \{ {( {{\text{x}}^{c} 0,{\text{y}}^{c} 0,{\text{t}}0} ),\, \left\langle {( {\upphi ^{c} ,{\text{t}}1^{c} } )} \right., \ldots ,( {\upphi ^{c} ,{\text{tn}}^{c} } )} \}$. ϕi^c^ calculation formula is expressed as:

1 $$ \upphi {\text{i}}^{{\text{c}}} = \arctan \frac{{yi^{c} - y0^{c} }}{{xi^{c} - x0^{c} }},\quad (1 \le {\text{i}} \le {\text{n,}}\quad 1 \le {\text{c}} \le {\text{k}}-1) $$

This allows the trajectory similarity function to:

2 $$ \upsigma ^{2} = \left( {\frac{{\mathop \sum \nolimits_{i = 1}^{n} \frac{{\Phi i^{2} - \Phi i}}{2\pi }}}{n}} \right)^{2} ,\quad\upsigma ^{2} \in 0,1 $$

From the function: The smaller σ^2^, the higher the corresponding candidate fake trajectory and the user's original trajectory similarity.

### Trie tree

#### **Definition 2**

(*Trie tree*) Trie tree, called a prefix tree or a dictionary tree for saving associated arrays. It can utilize all strings on the same node have the characteristics of public prefix, reducing the effect of query time and improving query efficiency.

#### **Definition 3**

**Figure 1:**
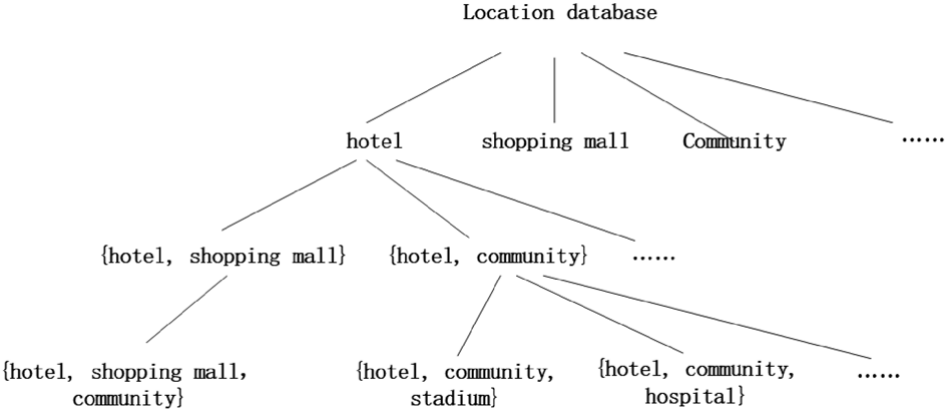
Position trie tree.

(*Position trie tree*^17^) The trie tree is accurately and quickly queries the location location of the same type or the same prefix when used in position data. When you check a location information, the more prefix you have, the more accurate the information found. For example, there are many hotels on the map. If we know the hotel is in the mall, then you can narrow the query to the hotel in the mall. If we can also know that the mall where this hotel is attached to a community, then the range of queries can be more accurate. Position trie tree is shown in Fig. 1.

#### **Definition 4**

(*Track trie tree*^18^) Track trie tree can get data such as traffic in the same time section. Fast and effective role in the statistics of the vehicle network data.

### Differential privacy

#### **Definition 5**

(*Differential privacy*^19^) It is a strong privacy protection technology. It mainly causes data distortion by adding noise in the data set input or output or the data set itself. Even if the attacker has a powerful background knowledge, it can also play an effective defense to achieve the purpose of privacy protection. This method ensures that the change in the output of is negligible. That is, even a single record in the data set is changed to cause privacy leaks of this record, this small risk of privacy leaks are also acceptable.

#### **Definition 6**

(*ε-Differential privacy*^19^) Set the probability of a collection of random algorithms A, *Pr* for all the final resulting query results. For any two adjacent data sets *D* and *D’*, and any subset *S* of *Pr*. If algorithm A is satisfied with the following, the algorithm A is called the *ε*-differential privacy:

3 $$ Pr\left( {A\left( D \right) \in S} \right) \le e^{\tau } \times Pr\left( {A\left( {D^{\prime} } \right) \in S} \right) $$

In the inequality, $S \subseteq Range( A )$. Data set *D* and *D’* have a maximum record different records. Parameter *ε* is used to measure the privacy protection budget.

#### **Definition 7**

(*Global sensitivity*^20^) For any function $f:D \to R^{d}$, Define $\Delta f = max_{{D,D^{\prime }\| f( D ) - f( {D^{\prime } } )\|_{1} }}$ is called global sensitivity of function f. In the equation: *d* is the dimension of the function output.

Global sensitivity means that the query function *f* is a maximum range that the query results may change when all possible adjacent data sets. Its metric depends on the L1 distance between *D* and *D’*. It is only related to the query function *f*, and is independent of the data set *D* itself. It is usually used to measure the amount of noise you need to add. The larger the global sensitivity of F, the larger the noise you need at the same time, and it is more likely to harm data utility.

### Disturbance mechanism

Index mechanisms and Laplace mechanisms are the two most widely noise mechanisms in differential privacy protection technology. The Laplace mechanism applies to privacy protection of numerical data, and the index mechanism applies to privacy protection for discrete data.

#### **Definition 8**

(*Laplace Mechanism*^21^) For any function $f{:}\,D \to R$, If the output result of the randomized algorithm A satisfies the formula (4), the algorithm A satisfies *ε*-differential privacy.

4 $$ {\text{A}}\left( {\text{D}} \right) = {\text{f}}\left( {\text{D}} \right) + {\text{Lap}}\left( {\frac{\Delta f}{\varepsilon }} \right) $$

In the equation: ${\text{Lap}}\left( {\frac{\Delta f}{\varepsilon }} \right)$ is the added Laplace noise.

According to the global sensitivity correlation, the probability difference formula is as follows:

5 $$ \frac{{Pr(f^{\prime} \left( D \right) + Lap\left( b \right) = y}}{{Pr(f^{\prime} \left( {D^{\prime} } \right) + Lap\left( b \right) = y}} \le \exp \left( \varepsilon \right) $$

Therefore: The smaller the privacy budget required for the privacy protection algorithm, the more Laplace noise needs to be added, the better the effect of privacy protection. But at the same time, the data distortion rate will be higher. So that the most suitable value should be selected based on the demand, not the pursuit of the privacy budget, the better.

## The basic idea of the algorithm

In order to protect the user's personal privacy and sensitive location information, a user LBS dual privacy protection algorithm based on orbital similarity is proposed. This algorithm first replaces the user's initial position based on the trajectory similarity algorithm. The sensitive position is then divided by the TRIE tree in the LBS database. Finally, the frequency of frequent times is disturbed by the Laplace mechanism by high to low rows.

**Figure 2:**
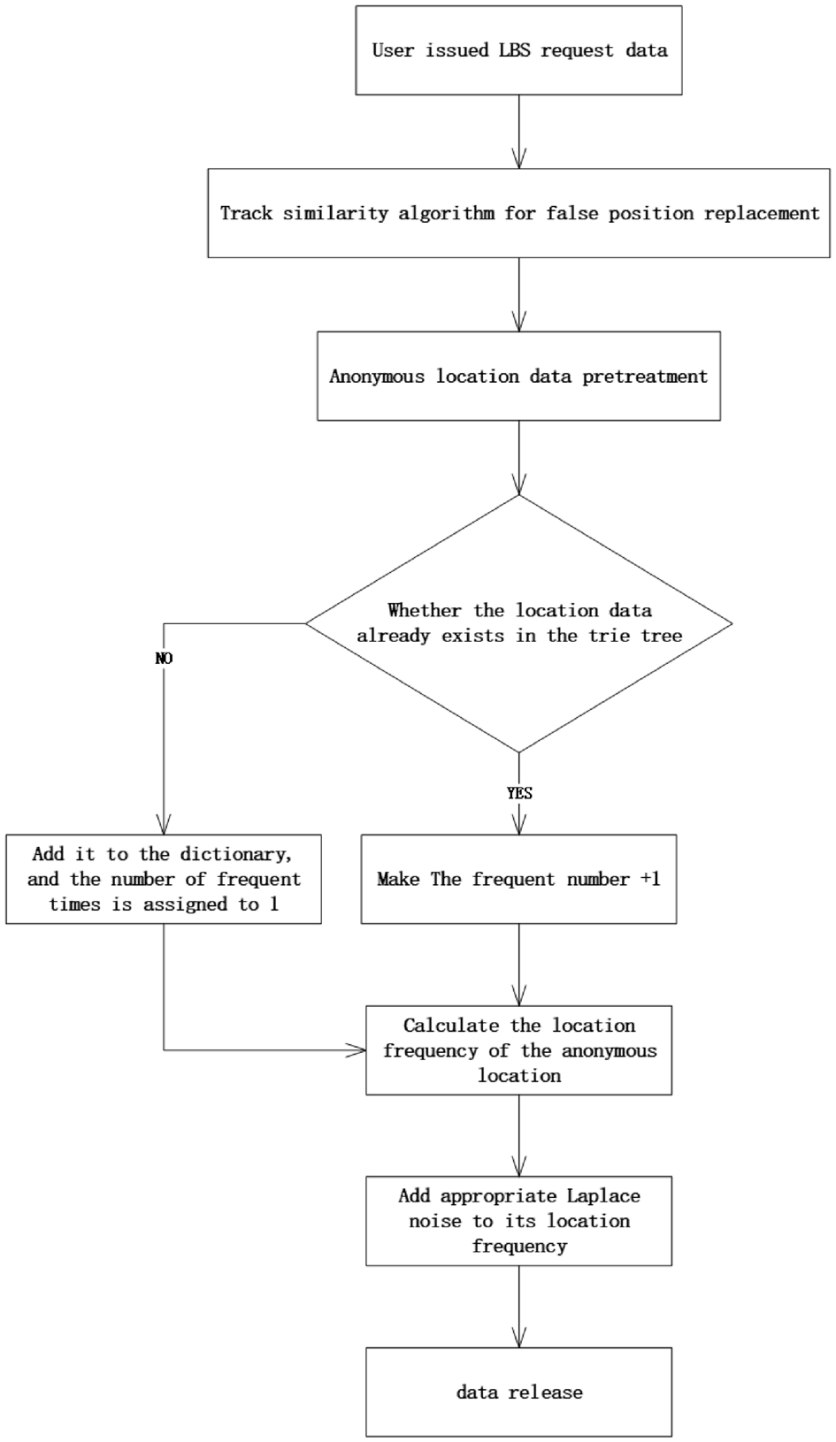
The algorithm flowchart.

The algorithm flowchart is shown in Fig. 2.

First of all, users are sending position service requests to the server. The original location and termination position of the location service request will be sent to the system at the same time. The system uses a false position algorithm of trajectory similarity to replace the request location with the optimal candidate. Then, the server processes the data of the fake location data to allocate the identifier {L1, L2, ……, Ln} for the fake location data. Assuming that a 30 × 30 grid is taken on the map, the information of the social venue in the grid is pre-processed by the location data as shown in Table 2.

**Table 2:** Location data pre-processing table.

Numbering	Storage unit information	Frequently
1	Shop	13
2	Community	34
3	Medical place	56
4	Stadium	8
5	Mall	26
6	Office building	22
…	…	…

After that, the collection content is converted into the location transaction database, and the content of the transaction database of the Trie tree is used. Location transaction database is shown in Table 3.

**Table 3:** Location transaction database.

Identifier	Position set	Frequently
L1–L18	{1}	13
L19–L29	{2}	34
L30–L37	{3}	56
L38–L42	{4}	8
L43–L47	{5}	26
L48–L70	{6}	22
L71–L84	{1, 2}	5
L85–L89	{1, 3}	3
L90–L93	{1, 4}	2
…	…	…

Finally, select frequently on the Trie tree.When the newly generated fake location is already in the dictionary, the number of frequently rises once. When the fake position is not found in the dictionary, the fake position is added into the dictionary, and the number of frequency number of the location is 1. After that, you only need to scan once to build the position transaction database and add the appropriate Laplace noise to the position frequency.

In this algorithm, the user first preloads the LBS request with its own real location data, read the starting position Q and the termination position z of the user request to generate trajectories. Sample space for Q and Z. Randomly generate K − 1 fake position in the sample space and randomly connect the false position points in Q and Z sample space to get K − 1 candidate trajectory. The similarity of each candidate trajectory and the original trajectory is calculated by the trajectory similarity algorithm, then select candidate trajectory with the lowest degree of origin. Alternate its starting position and termination location to q and z when the user initiates the LBS service request.

The algorithm pseudo code is as follows 
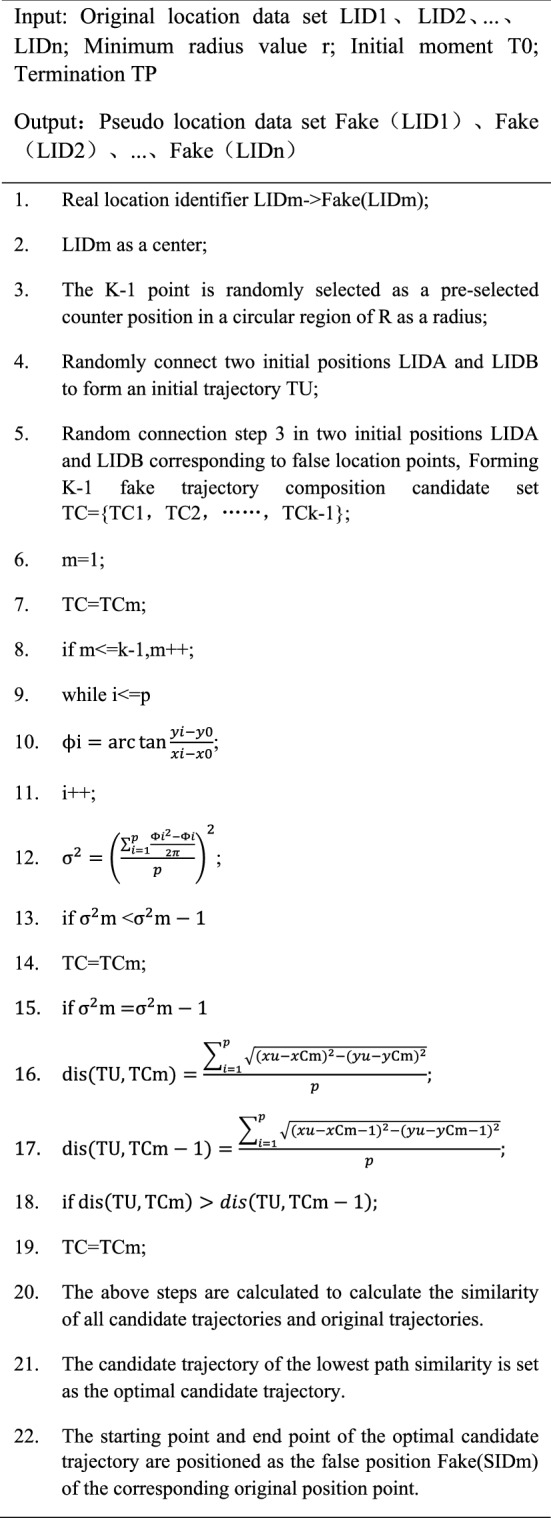
.

In the second part, preprocessing the LBS data set, assigns the number of position data in the collection, collecting shared location data into a collection of only numbered and location data. Transform this collection content into a location transaction database, and store the content of the location transaction database with the TRIE data structure. Finally, frequent mode analysis is performed on the TRIE tree, and the location frequency is added to the appropriate Laplace noise. This method has double protected by the user's own privacy and sensitive location information, which greatly improves the quality of privacy protection. The algorithm pseudo-code is as follows.
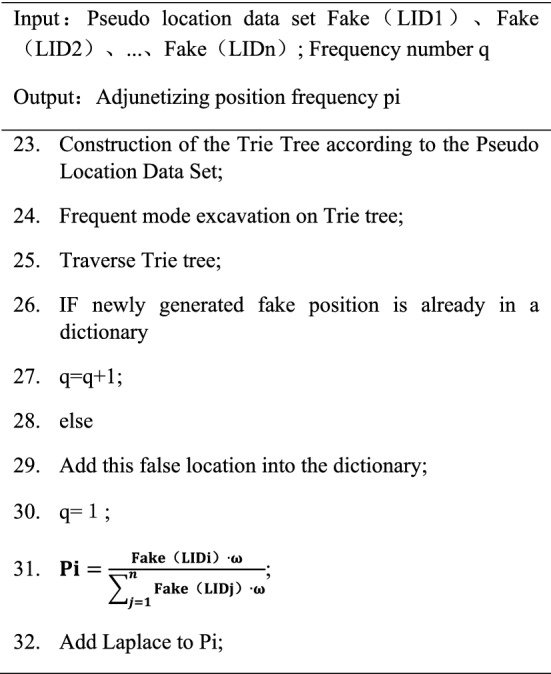


## Algorithm analysis

The first part of the algorithm (TSA, step 1–22 in the above algorithm), First, Calculate the angle of TC1 and TU at TI time relative to T0 time motion trajectory. The cycle calculation of the TC1 and TUs vary from respect to the motion trajectory of T0 with respect to T0 at all times. The similarity σ^2^m of all TCm and TUs is then calculated, and the similarity smaller trajectory is selected as the current optimal false trajectory. If there is a plurality of simplex minimum similarity, a candidate trajectory that is far from the original position of the candidate track start and the end point is selected according to the European distance formula. The algorithm can simultaneously encrypt the starting position and endpoint position at the same time, which greatly saves encrypting time and improves algorithm operating efficiency.

The second part of the algorithm (DPT, step 21–32 in the above algorithm). First, when the user performs a position service request, the user current location will be sent to the system. After the system uses the trajectory similarity algorithm to replace the user's current actual location using a false location, the server performs data pretreatment of false position data. The server will assign identifiers {L1, L2, …, Ln}, to the fake position data, convert the collection content into a location transaction database, and store the content of the location transaction database with TRIE tree. Finally, in the Trie tree, frequent location selection. When the newly generated fake position is already in the dictionary, the number of frequent times rises once. If you do not find the false position in the dictionary, add the false position to the dictionary, and the number of frequent times is only 1. After that, you can build a location transaction database and add the appropriate Laplace noise on the location frequency. The algorithm reduces data loss rate as much as possible under the privacy budget conditions required by the differential privacy. Effectively solve the problem of data distortion in the algorithm operation.

## Algorithm performance analysis

### Security analysis

In this article, in the process of initiating the LBS service request, the initiator was replaced by the optimal candidate instead of the LBS service query, so the query record information on the LBS server is the query information and identity information of the best candidate; The optimal candidates who continuously requested in different time intervals on the user's mobile trajectory are different. The attackers cannot infer their correlation through the intersection of an anonymous regional user set at different times.

Suppose the number of times the user inquiries on the mobile trajectory is m, and the number of candidates participating in the request is *n*_*i*_ (1 ≤ i ≤ m). Due to the different candidates during each query, candidates in different anonymous areas are independent of each other.

Assuming the probability of the attacker based on formula PID_U_ = D^T^*d*_*i*_ solve *d*_*i*_ is P(PID_U_) (*d*_*i*_ is the only N-dimensional vector allocated to each registered user, and meets $x^{{d_{i} }} = \rho$).The probability of intercepting the communication between the user U and the candidate, and the probability of decrypting the message is P(∂). Assume that the attacker obtains the user's registration request message to the server, then the probability of realizing the user tracking during the continuous query process ${\text{P}} = \prod\nolimits_{i = 1}^{m} {\frac{1}{{n_{i} }}P(\partial ) \times P({\text{PID}}_{{\text{U}}} )}$. P(∂) is equivalent to cracking the elliptical curve password system, which is not feasible in calculation.

P(PID_U_) equivalent to:Known PID_U_, find the value of *d*_*i*_ according to PID_U_ = D^T^*d*_*i*_. Among them, D is a N-dimensional column vector selected by the server randomly. The probability of solving *d*_*i*_ can be ignored. At the same time, the linear equation group $x^{{d_{i} }} = \rho$ has an endless solution, so the attacker cannot determine *d*_*i*_ through the matrix equation. All in all, you can ignore the probability of the attacker to learn about the true identity of the request. That is, the attacker cannot track the candidate through continuous query records, and then determine the true identity of the initiator. So the algorithm is safe and reliable.

### Practical analysis

The existing privacy protection algorithm based on prefixed trees needs to be traversed through all data sets, and then counts their position frequency, which greatly increases the workload of the algorithm. When the DPT algorithm in this article is added every time the new data is added, you only need to perform a traverse to know whether the data already exists in the dictionary. If the dictionary already exists, the frequency + 1. Otherwise, add it as an increase and add it into the data dictionary. After that, noise interference to the position frequency, that is, increase the degree of privacy protection, effectively improve the working efficiency of the algorithm, and reduce the working time of the algorithm.

DPTS algorithm communication mainly occurs between users and credible anonymous servers and LBS servers. Because the communication volume of these communication is constant level, it is recorded as O(C). First of all, the main communication content between users and credible anonymous servers is location service request and anonymous location set. Location service request communication volume is O(C), anonymous location set transmission depends on anonymous K, recorded as O(KC). Secondly, the credible anonymous server and the LBS server are mainly the transfer of false location information. The communication volume is still O(C). In summary, the overall communication volume of the algorithm is less than O (KC), the communication overhead is low, and it has high practicality.

### Time complexity analysis

The TSA algorithm is selected to select the fake position through the candidate set of the candidate, and the time complexity is O(n). So the time complexity of the TSA algorithm is O(n).

DPT algorithm is mainly divided into three stages: Phase 1 is to build Trie tree. If the character length of the location information is m, the finding time complexity of each location information is O(m). If there is an t location information, the time complexity of the Trie tree is O(*t*·*m*). Phase 2 to traverse Trie Tree. If you need to traverse N times, the time complexity of the process of finding the process of this location is O(*n*·*m*). Stage 3 to add noise at the position frequency, and the time complexity is O(n). Since the Trie tree only needs to be built once, it can be traveled directly, so the time complexity of the Trie tree can be ignored. So the overall time complexity of the DPTS algorithm is O(*n*·*m*).

## Results of the experiment

### Experiment environment and data set

The experiment is programmed by Python language. The experimental environment is Intel Core i5-1155G7CPU 2.5 GHz processor, 4 GB memory. Use the Linux operating system to install Hadoop for simulation experiments. Use the real data set and the Foursquare dataset^22^, select a total of 22,567 users, 27,898 locations and 1,467,543 user history check-in records. The configuration of the experimental parameter is shown in Table 4.

**Table 4:** Experimental parameters configuration table.

Parameter	Defaults	Ranges
*k*	7	[2, 10]
*f*	0.5	
*Ε*	0.02	[0.005, 0.4]
Historical location	27,898	
User number	22,567	
History sign-in record	1,467,543	

### Measurement criteria

This paper is analyzed from the algorithm from three aspects: Algorithm runtime, position entropy^20^ and data loss rate^21^:

1. The runtime of the algorithm affects the efficiency of the algorithm. In the same case of other conditions, the shorter the runtime of the algorithm, the higher the performance efficiency of the algorithm. Algorithms with execution efficiency can improve software running speed and effectively reduce costs.
2. Position entropy is the location information entropy. Location information entropy represents the probability that the location information appears within a particular area. After privacy protection, the lower the position entropy, the clearer the location information. Conversely, the higher the position entropy, the more blurred the location information, the better the effect of privacy protection. The calculation formula of information entropy is expressed as
  6 $$ {\mathbf{H}}\left( {\mathbf{x}} \right) = - {\mathbf{P}}\left( {{\mathbf{xi}}} \right){\mathbf{log}}\sum \left( {{\mathbf{2}},{\mathbf{P}}\left( {{\mathbf{xi}}} \right)} \right)\quad \left( {{\mathbf{i}} = {\mathbf{1}},{\mathbf{2}},..{\mathbf{n}}} \right) $$
  X is a random variable. In position entropy, x is represented as position point. P(x) represents the output probability function.
3. In differential privacy, the size of the added noise is not only affecting the degree of privacy, but also affects the loss rate of data. The larger the amount of noise added, the better the privacy protection, but excessive noise will cause data distortion to make the entire experimental data are not available.

### Results of the experiment

In order to verify the effectiveness of the algorithm, the TSA algorithm of this paper is compared to the IDLAS algorithm in reference^10^. In the case of the k value, two algorithms are compared, and the experimental results are shown below.

**Figure 3:**
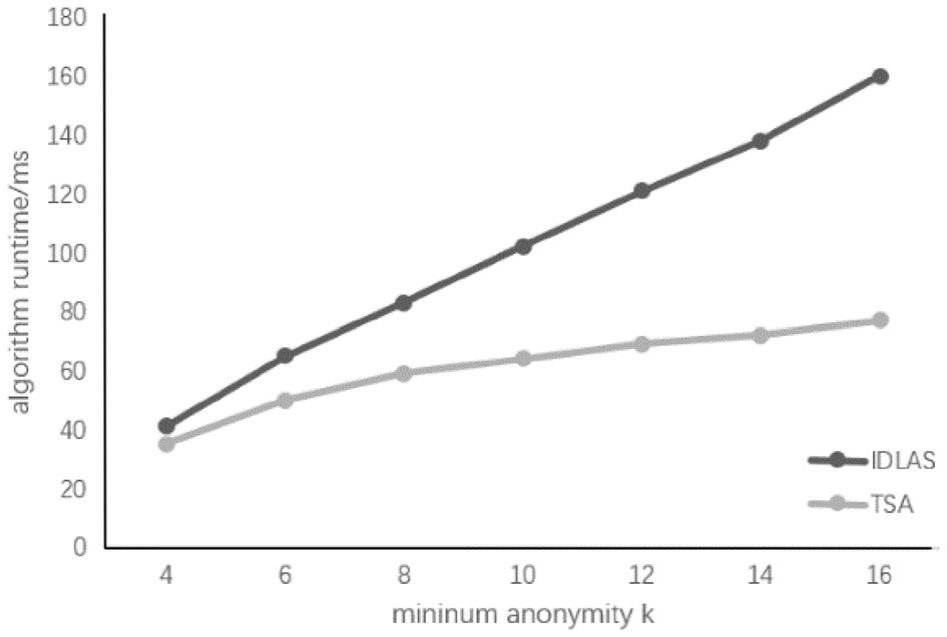
K value on algorithm runtime.

From the experimental results in Fig. 3: As the K value increases, the runtime of the algorithm is also increasing. Since the increase in K value results in an increase in the amount of experimental data, the complexity of the data operation is also increased, the longer the corresponding calculation time, the operation efficiency is lowered. Although the IDLAS algorithm enhances the confidence of anonymous data, the corresponding computation complexity is also larger. As the K value is increasing, the runtime of the algorithm will be more, and the efficiency of the IDLAS algorithm will be much smaller than the TSA algorithm.

**Figure 4:**
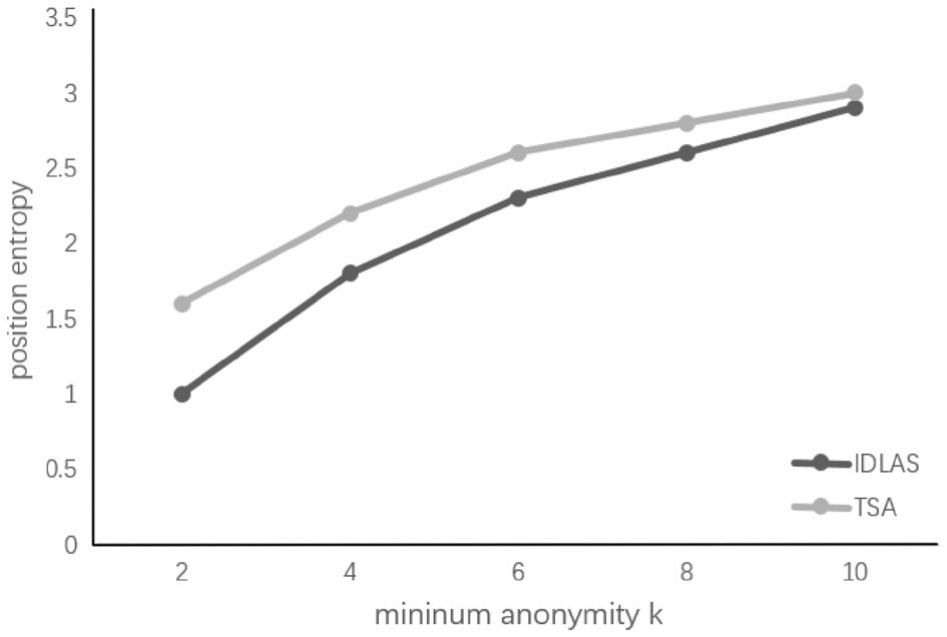
Location entropy.

As can be seen from the experimental results in Fig. 4, the position entropy increases as the K value increases. The experimental results show that when the K value is the same, the position entropy of the TSA algorithm is always superior to the position entropy of the IDLAS algorithm of^14^. Because the TSA algorithm pushes anonymous position via anonymous track, the encrypted false position is more blurred, which can better enhance the privacy protection.

**Figure 5:**
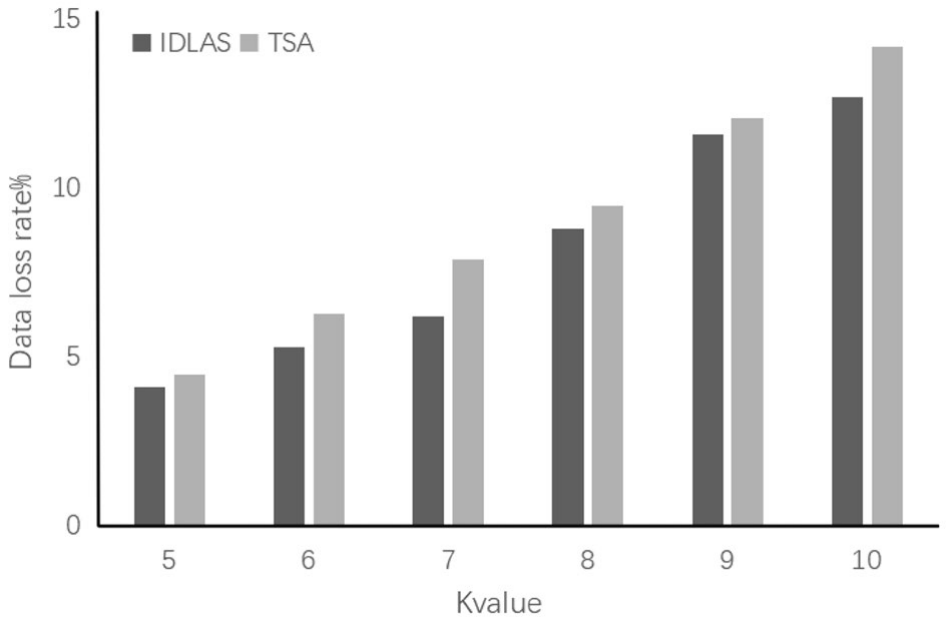
K value on data loss rate.

At different K values, data availability analysis experiments on IDLAS algorithms and TSA algorithms. As shown in Fig. 5, the larger the K value, the greater the data loss rate after pseudony mization, and the reduction of data availability. In the analysis experiment of the K value on the effect of privacy protection, the greater the K value, the better the privacy effect. However, if the selected K value is too big will cause data distortion. Therefore, the most suitable value should be selected according to other factors such as privacy protection budgets. After the experiment, when K = 7, the privacy protection effect and data availability of the TSA algorithm reached the best level.

**Figure 6:**
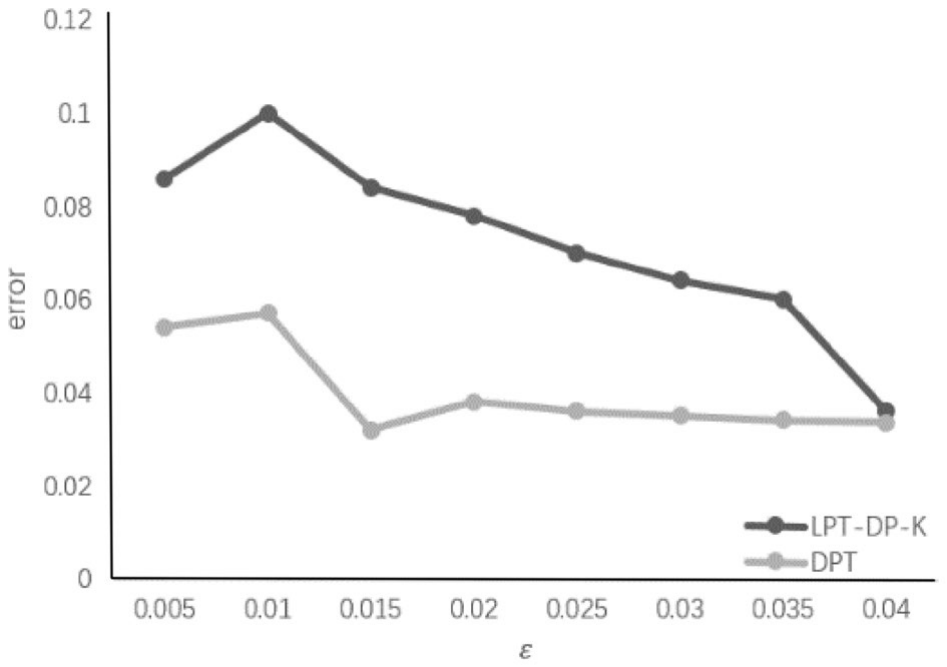
*ε* impact on error.

In order to determine the error caused by the addition of noise on the data, the experimental results of LPT-DP-K in References^17^ are compared to the DPT algorithm of this article. The experimental results are shown in Fig. 6, the more noise, the lower the privacy protection budget, the greater the error caused by the data set. Compared with the LPT-DP-K algorithm, the DPT algorithm used herein can reduce the difference privacy protection to the error, and effectively protect the sensitive location information while increasing the availability of data. Since the algorithm only adds noise at the frequency of position data, the amount of Laplace noise is not much influence on data distortion.

**Figure 7:**
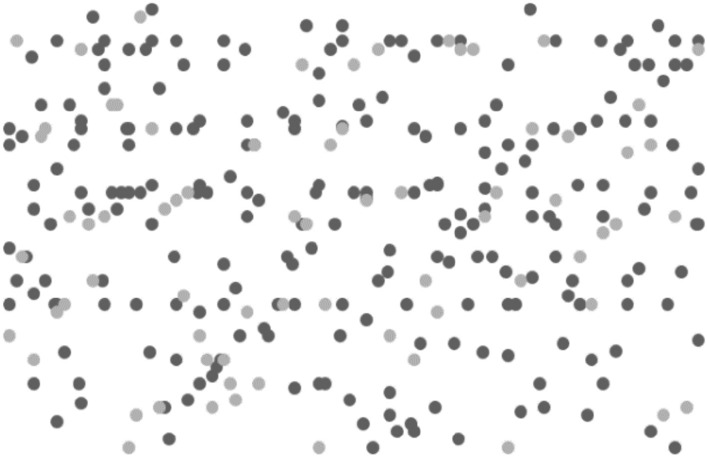
Before running the DPT algorithm.

**Figure 8:**
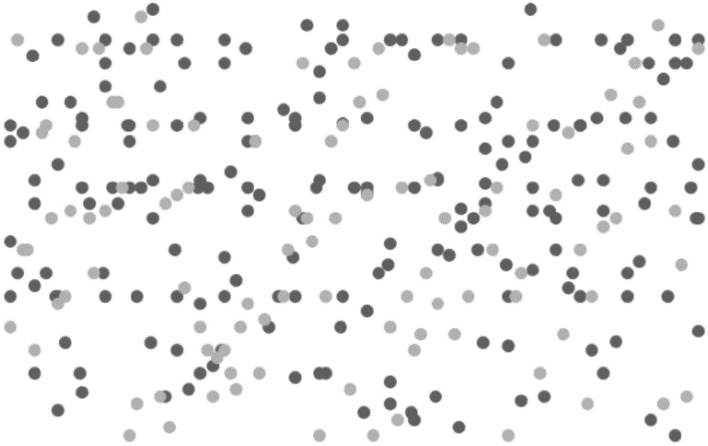
After running the DPT algorithm.

In order to more clearly analyze the degree of privacy protection of the DPT algorithm, this article selects 300 position data for research. Draw a comparison chart of sensitive positions before and after running the DPT algorithm, as shown in Figs. 7 and 8. Black in the figure is a sensitive location, gray is insensitive position.

Figure 7 shows that before the DPT algorithm is run, it can be seen that there are 73 sensitive locations at this time, 227 insensitive locations. Figure 8 is after running the DPT algorithm, the sensitive position is increased to 107, and the insensitive position becomes 193. Experiments show: After running the DPT algorithm, the sensitive position has changed after adding Laplace noise at frequent locations. Some originally non-sensitive locations transformed into sensitive locations, thereby implining the original sensitive position, enabling attackers unable to distinguish the real sensitive position and reach the desensitization of location information.

## Conclusion

The core idea of the user's LBS dual privacy protection scheme proposed in this paper is to add a fake position replacement algorithm based on the trajectory similarity-based fake position replacement algorithm and the frequent location selection algorithm based on TRIE tree. Combined with trajectory similarity, TRIE tree, frequent mode mining, and Differential privacy, a new algorithm for effectively protecting the LBS user personal information and sensitive location information is designed. This method is mainly divided into privacy protection. First, the fake position generated by the trajectory similarity is a false position replacement process for the user's original position before desensitization. Protect the user's personal privacy and sensitive location information, so that the degree of desensitivity is further increased. This method selects a trajectory that is the least similar to the user's original trajectory, as the optimal false trajectory by the track similarity algorithm. This method can obtain two false positions once, and there is almost no connection between the attributes of the false position and the original position. Attackers cannot infer the original location through attributes, so privacy protection has enhanced. By comparing with the existing relevant false position generating algorithm, the effectiveness of the fake position replacement algorithm based on the trajectory similarity is more advantageous. Second, combined with frequent mode excavation and Laplasuncing mechanism. Sensitivity to the generated false location is sorted by high to low in frequency. Add Laplace disturbance mechanism to the top k position data. Realize the secondary encryption of the location data that has been encrypted. The dual privacy protection algorithm based on the trajectory similarity is not only very high, but also further improving the effect of privacy protection. The application will continue to study the application of privacy protection in position trajectory.

## Data Availability

The datasets generated and analysed during the current study are available in the Foursquare repository, https://archive.org/download/201309_foursquare_dataset_umn online.
